# Association of ultra-processed food consumption with all-cause and cause-specific mortality: population-based cohort study

**DOI:** 10.3389/fnut.2026.1820451

**Published:** 2026-05-28

**Authors:** Ya-Dong Li, Le-Lan Gong, Yuan-Bin Jiang, Qiong Qin, Fei-Qiang Ren, Sheng-Qiang Qian, Wei Tan

**Affiliations:** 1 Department of Urology, Nanfang Hospital, Southern Medical University, Guangzhou, Guangdong, China; 2 Department of Proctology, The Second Affiliated Hospital of Kunming Medical University, Kunming, Yunan, China; 3 Department of Urology, The First Affiliated Hospital of Chongqing University of Chinese Medicine, Chongqing Traditional Chinese Medicine Hospital, Chongqing, China; 4 Department of Hepatobiliary Surgery, The First Affiliated Hospital of Chongqing Medical University, Chongqing, China

**Keywords:** all-cause mortality, cause-specific mortality, prospective cohort, risk factor, ultra-processed food

## Abstract

**Background:**

Ultra-processed food (UPF) consumption has been linked to adverse health effects, yet findings from prospective cohort studies on mortality remain inconsistent.

**Methods:**

This study included 82,221 participants from the Prostate, Lung, Colorectal and Ovarian (PLCO) Cancer Screening Trial. At baseline (1993–2001), dietary intake was assessed using the Baseline Questionnaire (BQ) and validated Dietary History Questionnaire (DHQ). UPF intake was defined according to the NOVA classification system. Daily food intake (g/day) was estimated from reported frequency and portion size, and energy and nutrient intakes were computed using the DietCalc analysis program. Mortality outcomes, including all-cause, cancer, circulatory system diseases, nervous system diseases, and other causes, were ascertained. Multivariable Cox models were used to estimate the associations between UPF intake and all-cause and cause-specific mortality.

**Results:**

During a median 17-year follow-up, 24,237 deaths occurred. No association was found between UPF intake and cancer mortality. Despite this, the highest UPF intake quarter, relative to the lowest, was associated with a 10% higher risk of all-cause mortality (HR: 1.10, 95% CI: 1.06–1.14), a 9% higher risk of circulatory system diseases mortality (HR: 1.09, 95% CI: 1.02–1.17), a 20% higher risk of nervous system diseases mortality (HR: 1.20, 95% CI: 1.06–1.37), and a 28% higher risk of mortality from other causes (HR: 1.28, 95% CI: 1.18–1.39). In the joint analysis of UPF intake and diet quality assessed by the Healthy Eating Index-2015 (HEI-2015), no consistent association was observed between UPF intake and mortality within most HEI-2015 quarters. However, among individuals in the highest quarter of UPF intake, those with the highest HEI-2015 scores had a significantly lower mortality risk than those with the lowest HEI-2015 scores, suggesting that higher diet quality may attenuate the adverse effect of high UPF consumption.

**Conclusion:**

Our study showed that a higher consumption of UPF was linked to increased mortality in all-cause, as well as in circulatory system diseases, nervous system diseases and other causes diseases.

## Graphical abstract

**Figure:**
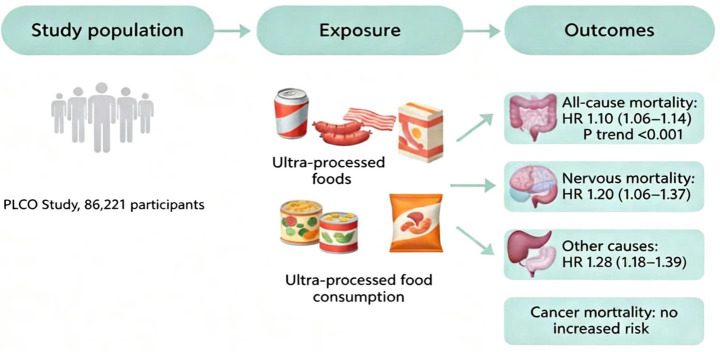

## Background

Ultra-processed foods (UPF) refer to industrial products formulated largely from food-derived substances, which are often chemically altered and combined with additives, containing minimal or no intact whole foods (1–3). UPF consumption has risen substantially worldwide due to their convenience, affordability, and palatability (4, 5). These products are reported to account for over half of the average person’s daily energy intake (5).

Compared to less processed foods, UPF tend to be high in total fat, added sugars, and salt, yet low in fiber and vitamins, thereby compromising dietary quality (3). In addition to their nutritional makeup, UPF may contain harmful substances, such as additives and contaminants formed during the processing. Because of the establishment of NOVA food classification system, growing epidemiological studies have reported the association between UPF and health outcomes, including diabetes, cardiovascular diseases and so on (6–8). Some studies have shown that there is a positive association between UPF intake and all-cause mortality (9–14). However, the relationship between UPF consumption and cause-specific death (e.g., cancer mortality) is debatable (7, 9, 15). However, few prospective cohort studies with follow-up exceeding 15 years have explored how UPF intake links to mortality from all-cause or specific-causes, while considering impact of diet quality.

In this study, combined with dietary quality, we evaluated the relationship of total and subgroup UPF intake with mortality from all-cause or specific-causes, including cancer, circulatory system diseases (such as ischemic heart disease and cerebrovascular), nervous system diseases (such as Alzheimer’s disease, and Parkinson’s disease) and other diseases (such as digestive diseases, respiratory diseases, and infectious disease) in a large US population.

## Methods

Here is a brief summary of the materials and methods; more detailed information is recorded in Supplementary material entitled “Supplementary--Materials and Methods”.

### Selection of the study population

**Figure 1:**
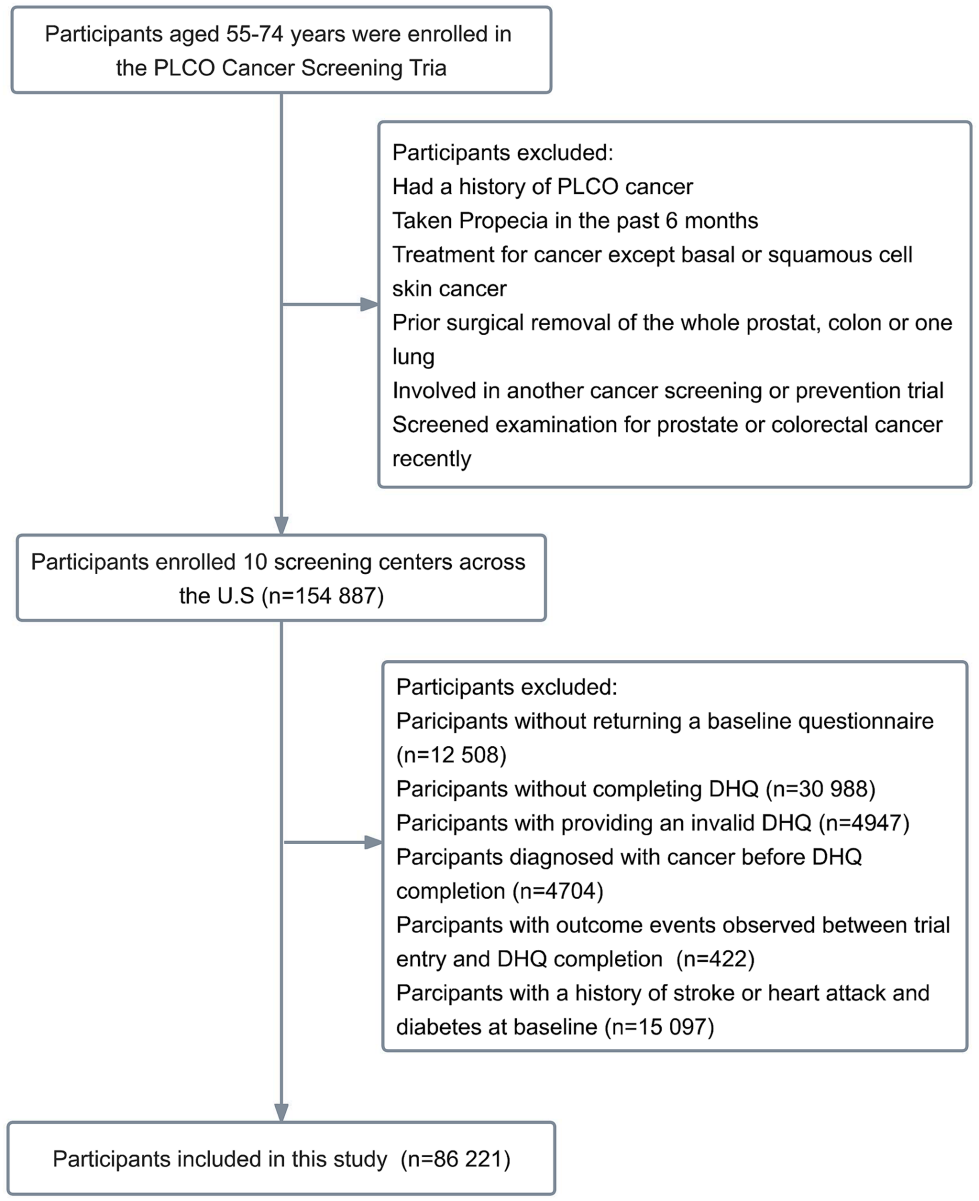
The flow chart of identifying individuals eligible for our study.

The PLCO (Prostate, Lung, Colorectal and Ovarian) Cancer Screening Trial recruited approximately 155,000 individuals aged 55–74 from 1993 to 2001, collecting participant information through standardized questionnaires. Participants’ information was collected using standardized questionnaires, including the Baseline Questionnaire (BQ) and the validated Diet History Questionnaire (DHQ), to quantify dietary nutrient and food group intakes. After exclusions, we included 86,221 participants (39,494 males and 46,727 females). The flowchart was presented in Figure 1.

### Assessment of UPF consumption

Base on NOVA classification, we calculated the percentage (%) of UPF relative to the total daily consumed weight of all food and beverages (g/day).

We specifically employed the weight ratio instead of the energy ratio to encompass both no energy provided UPFs (such as soft drinks) and processing-related non-nutritional aspects (such as additives).

### Assessment of covariates

We incorporated and assessed age, race/ethnicity, education, family cancer history, smoking, physical activity, hypertension history, aspirin intake, alcohol intake, body mass index (BMI), and energy intake.

### Ascertainment of outcomes

All-cause mortality served as the primary outcome in this study. The secondary outcomes were cause-specific mortality, including deaths from cancer (ICD-9 codes 140–207), circulatory system diseases (ICD-9 codes 390–459, 430–438), nervous system diseases (ICD-9 codes 320–389) and other diseases (ICD-9 codes 460–519, 240–279, 001–139, 580–629, 800–999). In this study, death certificates and family reports were the sources of further confirmed outcomes.

### Statistical analysis

**Figure 2:**
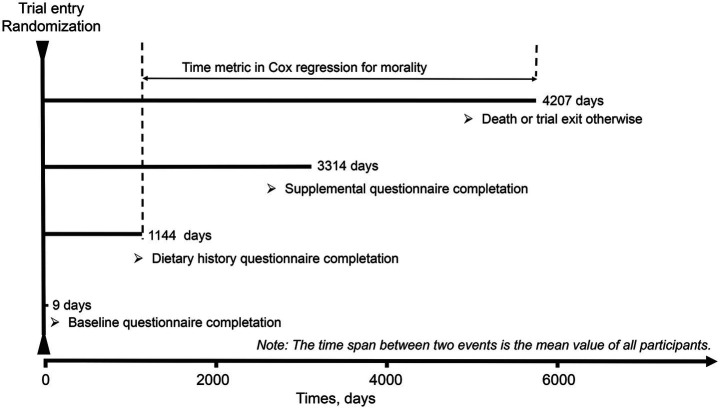
The timeline and follow-up scheme of our study. The baseline point in this study was set at the date of diet history questionnaire completion.

In this study, we conducted Cox proportional hazards regression on the relationship between UPF consumption and all-cause as well as cause-specific mortality. Race/ethnicity and age at DHQ completion were adjusted in model 1. Model 2 was further adjusted for established risk factors for mortality, including alcohol intake (g/day, continuous), BMI (kg/m^2^, continuous), education, smoking, energy consumption (kcal/day, continuous), family cancer history, aspirin use and hypertension history. In these models, participants contributed person-time from DHQ completion until death, or 31 December 2009, whichever came first (Figure 2). To test nonlinearity, we evaluated the null hypothesis that the second spline term’s regression coefficient equals zero. Furthermore, we jointly categorized participants by quarters of Healthy Eating Index-2015 (HEI-2015) and UPF consumption, using those in the highest HEI and lowest UPF quarters as the reference group.

Subgroup analyses were performed according to age (<65 vs. >65 years), BMI (<25 vs. >25 kg/m2), trial group (screening vs. control groups), smoking status (current or former vs. never), and alcohol consumption (<median vs. ≥median). Sensitivity analyses were conducted to assess the robustness of these findings. All statistical analyses were conducted using R software version 4.3.1. Two-sided *p* < 0.05 was deemed statistically significant.

## Results

### Baseline characteristics

During a median of 17 years of follow-up, we documented 24,237 deaths (10,702 females and 13,535 males), among which 7,116 deaths due to cancer, 6,993 deaths due to circulatory system diseases, 3,926 deaths due to nervous system diseases, and 5,504 deaths due to other diseases (including respiratory and digestive diseases, etc.). Baseline characteristics stratified by sex-specific quarters of dietary UPF proportion are summarized in Table 1. Participants with higher UPF consumption tended to be younger, had a greater likelihood of smoking, and exhibited higher BMI. They also showed lower intakes of fruits, vegetables, whole grains, and dietary fiber, along with reduced physical activity and poorer HEI-2015 scores. The distribution of dietary UPF proportion in the study population is provided in Supplementary Figure 1.

**Table 1:** Baseline characteristics of the PLCO study population according to sex specific quarters of ultra-processed food consumption (*n* = 86,221).

Characteristics	Quarters of ultra-processed food consumption^1^				
	All participants	Q1 (*n* = 21,556)	Q2 (*n* = 21,555)	Q3 (*n* = 21,554)	Q4 (*n* = 21,556)
Age, years	65.3 (5.7)	66.0 (5.7)	65.7 (5.7)	65.2 (5.7)	64.2 (5.5)
Body mass index (kg/m^2^)	27.0 (4.6)	26.0 (4.2)	26.6 (4.4)	27.2 (4.6)	28.0 (5.1)
Physical activity (min/week)^2^	126.7 (122.7)	135.9 (127.4)	127.4 (120.9)	123.8 (119.9)	119.7 (121.9)
Males	39,494 (45.8%)	9,874 (45.8%)	9,873 (45.8%)	9,873 (45.8%)	9,874 (45.8%)
Family history of cancer, *n* (%)	48,598 (56.4%)	12,103 (56.1%)	12,220 (56.7%)	12,176 (56.5%)	12,099 (56.1%)
History of hypertension, *n* (%)	24,727 (28.7%)	5,557 (25.8%)	5,934 (27.5%)	6,293 (29.2%)	6,943 (32.2%)
Aspirin user, *n* (%)	37,471 (43.5%)	9,389 (43.6%)	9,512 (44.1%)	9,414 (43.7%)	9,156 (42.5%)
Racial/ethnic group, *n* (%)					
Non-Hispanic White	78,926 (91.5%)	19,464 (90%)	19,944 (93%)	19,893 (92%)	19,625 (91%)
Non-Hispanic Black	2,515 (2.9%)	313 (1.5%)	428 (2.0%)	661 (3.1%)	1,113 (5.2%)
Hispanic	1,198 (1.4%)	311 (1.4%)	304 (1.4%)	280 (1.3%)	303 (1.4%)
Other race/ethnicity ^3^	3,582 (4.2%)	1,468 (6.8%)	879 (4.1%)	720 (3.3%)	515 (2.4%)
Body mass index, *n* (%)					
Underweight (<18.5 kg/m^2^)	595 (0.7%)	225 (1.0%)	147 (0.7%)	117 (0.5%)	106 (0.5%)
Normal (18.5–24.9 kg/m^2^)	30,271 (35.1%)	9,316 (43.2%)	8,037 (37.3%)	7,037 (32.6%)	5,881 (27.3%)
Overweight (25–29.9 kg/m^2^)	37,334 (43.3%)	8,833 (41.0%)	9,403 (43.6%)	9,672 (449%)	9,426 (43.7%)
Obese (>30 kg/m^2^)	18,021 (20.9%)	3,182 (14.8%)	3,968 (18.4%)	4,728 (21.9%)	6,143 (28.5%)
Educational degree, *n* (%)					
College below	16,514 (19.2%)	4,703 (21.8%)	4,186 (19.4%)	3,977 (18.5%)	3,648 (16.9%)
College graduate	15,450 (17.6%)	4,100 (19.0%)	4,049 (18.8%)	3,803 (17.6%)	3,498 (16.2%)
Postgraduate	54,257 (62.9%)	12,753 (59.2%)	13,320 (61.8%)	13,774 (63.9%)	14,410 (66.8%)
Smoking status, *n* (%)					
Never	42,481 (49.3%)	9,325 (43.3%)	10,451 (48.5%)	11,107 (51.5%)	11,598 (53.8%)
Current	8,013 (9.3%)	2,333 (10.8%)	1,973 (9.2%)	1,727 (8.0%)	1,980 (9.2%)
Former	35,727 (41.1%)	9,898 (45.9%)	9,131 (42.4%)	8,720 (40.5%)	7,978 (37.0%)
Alcohol intake, g/d	9.7 (25.3)	15.5 (38.4)	9.9 (20.8)	7.8 (17.6)	5.8 (16.8)
Energy intake from diet, kcal/day	1,736 (733)	1,585 (693)	1,723 (689)	1,805 (731)	1,832 (789)
Healthy Eating Index-2015	66.6 (9.7)	71.2 (9.1)	67.9 (8.8)	65.5 (9.0)	61.8 (9.4)
Food consumption					
Whole grain (g/day)	59.9 (58.9)	67.0 (67.5)	61.7 (57.3)	59.6 (55.5)	51.3 (53.1)
Vegetable (g/day)	283 (185)	304 (217)	290 (180)	283 (173)	254 (161)
Fruit (g/day)	274 (218)	307 (253)	278 (204)	270 (201)	237 (193)
Red Meats (g/day)	60.7 (51.5)	46.6 (41.5)	60.9 (50.3)	65.9 (53.0)	69.3 (57.1)
White Meats (g/day)	50.8 (47.9)	48.0 (46.6)	51.5 (46.3)	52.9 (47.8)	50.6 (47.9)
Nutrient intake					
Fat (g/day)	62.4 (33.4)	51.3 (26.8)	62.4 (31.7)	67.0 (34.4)	69.0 (36.8)
Carbohydrate (g/day)	221 (91.1)	203 (83.8)	217 (83.3)	229 (89.4)	236 (103)
Protein (g/day)	66.2 (29.9)	61.5 (27.2)	67.2 (29.5)	69.1 (30.8)	67.0 (31.4)
Cholesterol (mg/day)	207 (133)	176 (119)	209 (128)	221 (136)	223 (142)
Dietary fiber (g/day)	17.9 (8.4)	18.5 (9.4)	18.2 (8.2)	18.2 (8.1)	16.9 (7.7)
Sodium (mg/day)	2,707 (1,195)	2,401 (1,031)	2,725 (1,148)	2,864 (1,237)	2,839 (1,290)

*Values are mean ± SD or numbers (percentages).

^1^Quarters of proportion of ultra-processed food intake in total quantity of food consumed. Sex specific cut-offs for quarters of ultra-processed proportions were 7.7%, 12.3%, and 19.1% in men and 6.5%, 10.7%, and 17.6% in women. Additionally, in the overall study population (regardless of sex), the mean UPF intake was 17.2%. ^2^Total time of moderate-to-vigorous physical activity per week. ^3^Other race/ethnicity = Asian, Pacific Islander or American Indian.

### Associations between UPF intake and mortality

The results of mortality across quarters of UPF intake are presented in Table 2. Adjustment for age, sex, and marital status indicated a positive relationship between UPF consumption and mortality outcomes, which was notably weakened after further multivariable adjustment. Compared with the lowest quarter, participants in the highest quarter were associated with increased risks of all-cause mortality (HR: 1.10, 95% CI: 1.06–1.14; *p* for trend < 0.001), circulatory disease mortality (HR: 1.09, 95% CI: 1.02–1.17; *p* for trend < 0.001), nervous system diseases mortality (HR: 1.20, 95% CI: 1.06–1.37; *p* for trend < 0.001), and mortality from other causes (HR: 1.28, 95% CI: 1.18–1.39; *p* for trend < 0.001). However, we found no associations for deaths due to cancer diseases.

**Table 2:** Hazard ratios and 95% confidence intervals for mortality according to ultra-processed food (UPF) consumption.

Patient group	Sex specific quarters of proportion of ultra-processed food consumption				
	Q1 (*n* = 21,556)	Q2 (*n* = 21,555)	Q3 (*n* = 21,554)	Q4 (*n* = 21,556)	*p*_trend_
All-case mortality					
Model 1^1^	1.00 (reference)	0.98 (0.95–1.02)	1.02 (0.99–1.06)	1.13 (1.09–1.17)	<0.001
Model 2^2^	1.00 (reference)	0.97 (0.94–1.01)	1.01 (0.97–1.05)	1.10 (1.06–1.14)	<0.001
Cancer mortality					
Model 1^1^	1.00 (reference)	0.98 (0.92–1.05)	0.95 (0.89–1.02)	0.99 (0.92–1.05)	0.492
Model 2^2^	1.00 (reference)	0.98 (0.92–1.05)	0.95 (0.89–1.01)	0.98 (0.92–1.06)	0.456
Circulatory mortality					
Model 1^1^	1.00 (reference)	0.97 (0.91–1.04)	1.09 (1.02–1.16)	1.16 (1.09–1.25)	0.007
Model 2^2^	1.00 (reference)	0.95 (0.89–1.01)	1.05 (0.98–1.12)	1.09 (1.02–1.17)	0.001
Nervous mortality					
Model 1^1^	1.00 (reference)	1.06 (0.94–1.19)	1.06 (0.94–1.19)	1.19 (1.05–1.35)	0.009
Model 2^2^	1.00 (reference)	1.06 (0.94–1.19)	1.06 (0.94–1.20)	1.20 (1.06–1.37)	0.008
Other mortality					
Model 1^1^	1.00 (reference)	1.03 (0.95–1.11)	1.10 (1.02–1.19)	1.32 (1.23–1.42)	<0.001
Model 2^2^	1.00 (reference)	1.02 (0.94–1.10)	1.08 (1.00–1.17)	1.28 (1.18–1.39)	<0.001

^1^Age (years), sex (male, female), race (non-Hispanic White, non-Hispanic Black, Hispanic, and other race/ethnicity), and marital status. ^2^Adjusted for covariates in model 1 plus smoking status [current (>20 cigarettes/day, 10–20 cigarettes/day, <10 cigarettes/day), former (stop smoking >15 years, stop smoking ≤15 years), never], alcohol consumption (g/day), body mass index (kg/m^2^), aspirin use (yes, no), history of hypertension (yes, no), family of cancer (yes, no), energy intake from diet (kcal/day), physical activity, and educational level.

**Figure 3:**
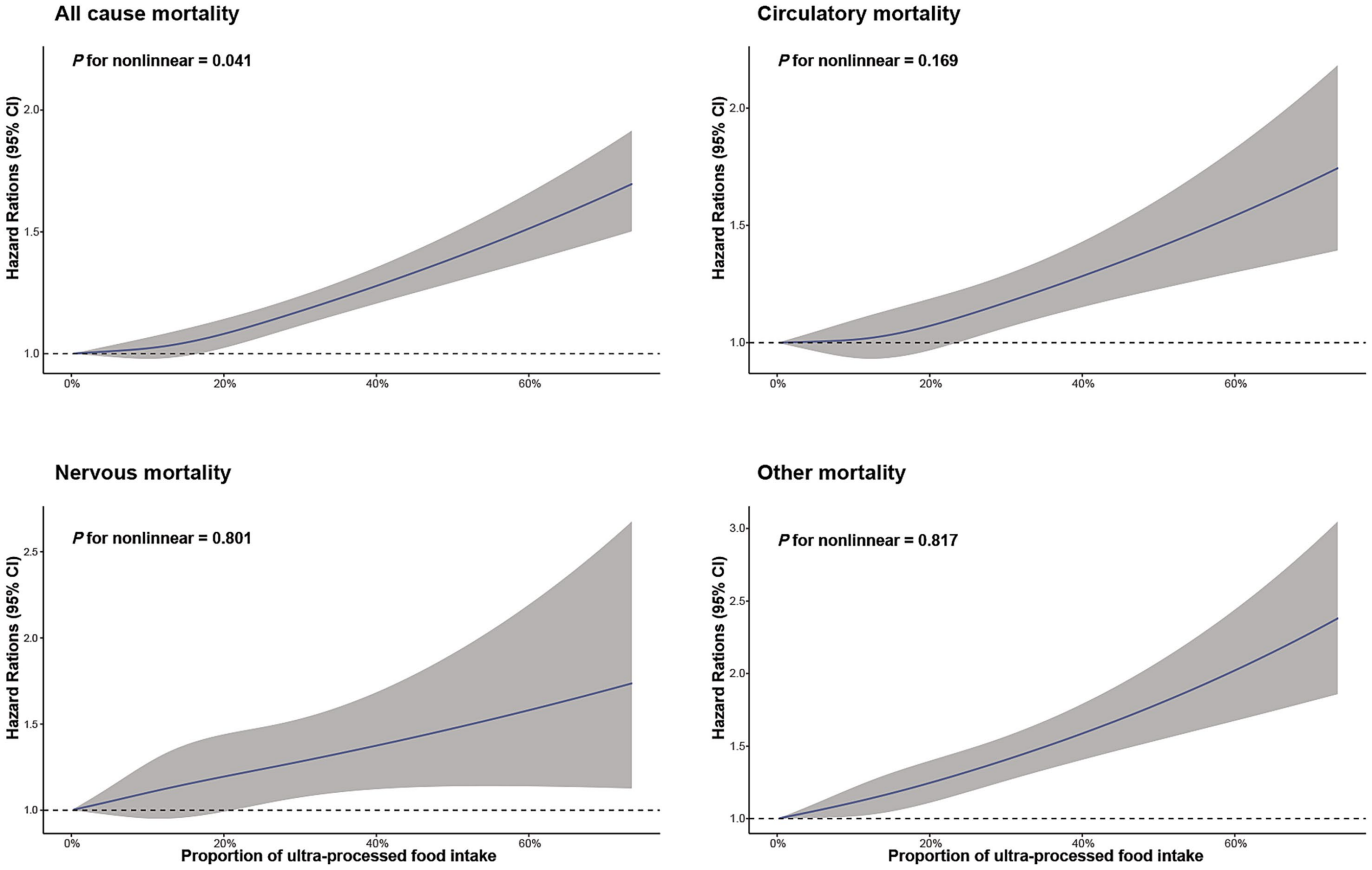
Nonlinear dose–response analyses on energy-adjusted ultra-processed food consumption and all cause, circulatory, nervous and other mortality in the whole study population.

**Figure 4:**
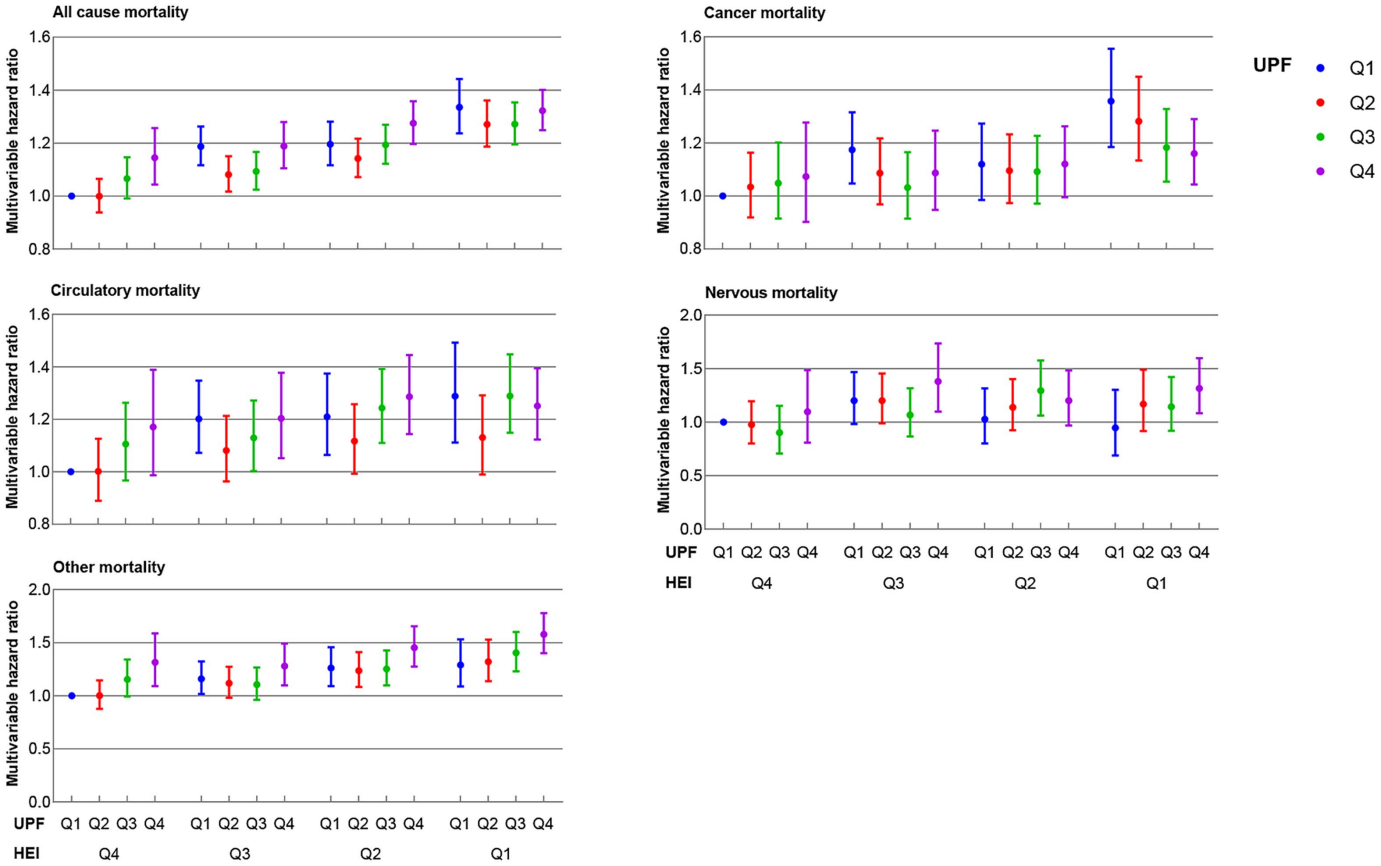
Joint analysis for mortality according to quarters of ultra-processed food (UPF) consumption and quarters of Healthy Eating Index-2015 (HEI) score. Participant were classified by quarters of UPF intake and HEI score, resulting in 16 groups. Using this combined variable as the exposure, we examined the association with mortality, with the highest HEI quarter (Q4) and lowest UPF quarter (Q1) as the reference. Points represent hazard ratio estimates; error bars show 95% confidence intervals.

The linearity assumption between UPF and the risk of mortality was analyzed by the restricted cubic spline regression (all *p* nonlinearity > 0.05) (Figure 3). Then we conducted a joint analysis of UPF intake and HEI-2015 score (Figure 4). The results indicated that while UPF intake had no distinct impact on mortality when stratified by HEI-2015 quarters, a higher HEI-2015 score consistently exhibited a protective effect against mortality regardless of the UPF consumption. Furthermore, main food groups which contributed to UPF intake were soft drinks and cereals were the dominant contributors, accounting for 48.1 and 16.3% respectively, succeeded by ultra-processed fruits and vegetables (13.1%) and dairy (7.05%) (Supplementary Figure 2). Furthermore, a significant increase in all-cause mortality risk was observed with higher consumption of soft drinks (HR: 1.05 95% CI: 1.01–1.10, *p* for trend = 0.011) and cereals (HR: 1.05 95% CI: 1.01–1.10, *p* for trend = 0.005) in the Supplementary Table 4.

We conducted subgroup analyses based on major risk factors, the association between UPF intake and all-cause mortality appeared to be stronger among participants with higher BMI (*p* for interaction = 0.011). However, no significant interactions were observed for age, sex, smoking status, or alcohol consumption (Supplementary Table 5). In sensitivity analyses, this association of UPF intake with all-cause mortality remained significant. However, this associations were attenuated when we adjusted for HEI-2015 scores in model 2 (Supplementary Table 6). Furthermore, we repeated the primary analysis using either the percentage of energy intake or the absolute quantity to measure UPF intake and observed similar results (Supplementary Table 6).

## Discussion

In this large, prospective, multicenter cohort involving 86,221 participants from US population, higher consumption of UPF was found to be associated with modestly higher all-cause mortality. In contrast, it was not linked to cancer-specific mortality. These associations differed by UPF source. Cereals and soft drinks were consistently linked to higher all-cause, as well as to cause-specific mortality. Notably, these associations were attenuated after adjusting for overall dietary quality.

### Interpretation and comparison with other studies

Previous research has indicated a potential link between UPF consumption and mortality risk. A meta-analysis demonstrated that the individuals with the highest UPF intake had a higher risk of all-cause mortality than those with the lowest intake (HR: 1.21, 95% CI: 1.13–1.30). However, apart from the mortality rates caused by cancer and cardiovascular diseases, limited evidence is available regarding other cause-specific mortality outcomes. In recent years, multiple studies have supported the association of UPF intake with both all-cause and disease-specific mortality (9–14). Compared with most existing studies, our analysis included the same well-established prospective cohorts with substantially larger sample sizes and extended follow-up, while simultaneously adjusting for overall dietary quality. These strengths enhanced the robustness of our findings and provided stronger evidence supporting this association.

In two large US cohorts comprising female nurses and male health professionals with more than 48,000 deaths documented over three decades, higher UPF intake was modestly associated with increased all-cause and non-cancer, non-cardiovascular mortality, particularly for processed meat products, sweetened beverages, dairy-based desserts, and ultra-processed breakfast foods (16). However, no significant relationship was found with cancer mortality. Among existing cohort studies, two different cohorts both reported that UPF consumption was positively linked to all-cause, cardiovascular, and respiratory mortality, but showed no association with cancer mortality (9, 15). A cohort study including 38,148 US adults similarly found that higher UPF intake was linked to increased mortality of all-cause and cardiovascular disease (17). Another study focusing on cardiovascular diseases also confirmed that UPF intake was linked to an increased cardiovascular mortality (6). Besides, a large multinational prospective cohort involving 428,728 participants likewise found positive correlations between UPF consumption and mortality of all-cause, as well as circulatory and digestive system diseases mortality (18).

However, some findings remained controversial. A study involving an older US population found that UPF intake was linked to higher all-cause and cancer mortality, but showed no association with cardiovascular mortality (7). Another study focusing on specific cancer types suggested that high UPF intake increased mortality from lung and prostate cancers (19). In the research related to the Southern Community Cohort, it was found that higher UPF intake was not linked to all-cause, cancer, coronary heart disease (CHD), or stroke mortality, but showed a significant association with increased diabetes mortality (20). But another study of the same prospective cohort found that participants with higher intake of UPF had an elevated risk of liver cancer (21). These discrepancies in findings might be attributable to differences in the baseline characteristics of patient populations across studies, as well as the influence of confounding factors such as lifestyle and economic conditions in different regions. Consequently, further high-quality research is required to substantiate these findings. In further subgroup analyses, we found that intake of ultra-processed cereals was positively associated with all-cause mortality and nervous system diseases mortality, while intake of ultra-processed soft drinks was positively linked to all-cause mortality and circulatory system diseases mortality. Analysis of the proportion of different food types within the UPF category revealed that ultra-processed cereals and soft drinks were the most significant contributors.

Currently, some countries have begun implementing national policies to reduce UPF consumption, including taxing sugar-sweetened beverages and implementing restrictions on the marketing of UPF to children (22). Our findings may offer valuable insights for shaping such policies and dietary guidelines.

Stratified analysis showed that the apositive association between UPF intake and all-cause mortality was stronger in individuals with higher BMI. Given that obesity itself is an independent risk factor of various diseases, including cardiovascular, metabolic diseases, and cancer (23–26), BMI may interact synergistically with UPF intake, potentially amplifying their adverse effects.

The HEI-2015 is a measure of diet quality based on the 2015–2020 Dietary Guidelines for Americans, which emphasize key food groups and nutrients (27). After adjusting for overall diet quality using the HEI-2015, we found that higher UPF intake was still associated with an increased risk of mortality, even among those with high HEI scores. Similarly, a study investigating different levels of adherence to the Mediterranean diet and UPF intake found that, for the same level of Mediterranean diet adherence, higher UPF intake was linked to increased all-cause mortality (28). This suggested that high UPF intake might diminish the protective effects of a healthy diet, highlighting the importance of reducing UPF consumption as a key public health recommendation.

Several potential mechanisms might explain the link between UPF intake and mortality. Firstly, high consumption of UPF often leads to a reduced intake of non-UPF (29), and lower overall diet quality (30). Secondly, UPF generally lacks fiber, protein, and micronutrients, and is high in energy, added sugar and sodium (22), which may result in atherosclerosis, hypertension, diabetes and other conditions (31, 32). Thirdly, various additives in UPF have been linked to increased mortality (33). For example, flavors can contribute to body weight gain and obesity by enhancing hedonic eating and disrupting flavor-nutrient learning (34). The development of glucose intolerance in humans can be driven by sweeteners, which reshape the intestinal microbiota’s composition and function (35). Fourthly, toxic chemicals may be produced during the production progress (36). Fifthly, some harmful chemicals in product packaging can migrate into food during the preservation of UPF (37, 38).

### Strengths and limitations of this study

The strengths of our study included its prospective design, large sample size (82,221 participants, 24,237 deaths), and long follow-up period (median 17 years). However, several limitations should be considered. First, dietary information was collected only at the beginning of the study, and potential changes in diet that may occurred during the follow-up were not assessed, which might have influenced the results. Second, as an observational study, the possibility of residual confounding remains. Although we used various methods to account for potential confounders, completely eliminating the influence of unmeasured or unmeasurable factors was impossible. Third, our findings were based on the specific population studied, their applicability to other ethnicities or populations with different baseline characteristics required further validation. Fourth, it should be noted that although we examined the relationship between UPF consumption and mortality of circulatory system diseases (including ischemic heart disease and cerebrovascular disease), previous studies had mostly focused on cardiovascular diseases. To our knowledge, only one previous study had assessed the relationship between UPF and circulatory system disease mortality, and the specific composition of circulatory system diseases in that study was not entirely consistent with ours (18). Therefore, caution is needed when comparing our findings with those of previous studies that defined cardiovascular or circulatory system diseases differently. Fifth, the mean proportion of UPF intake in our study population was 17.2%, which differed from the reported UPF intake in previous studies. This difference might be attributed to several factors, including the older age of our participants (mean age 65.26 ± 5.68 years), as dietary patterns and food choices often differ between older and younger adults. Additionally, variations in dietary assessment instruments and the classification criteria for UPF across studies might contribute to heterogeneity in estimated intake levels. Furthermore, the baseline dietary data were collected between 1993 and 2001, a period during which the availability and consumption of UPF were likely lower than in more recent years. Therefore, our findings may not be directly generalizable to contemporary populations, and future studies are necessary to examine whether similar relationship hold in populations exposed to different dietary patterns.

## Conclusion

In this study, UPF consumption was linked to slightly increased all-cause mortality. Overall, the mortality associations for UPF intake were more modest than for diet quality, but varied by subgroup. Cereals and soft drinks consistently demonstrated the strongest associations with mortality. These findings supported limiting certain types of UPF for long-term health. Further researches are necessary to refine UPF classification and to confirm these associations in other populations.

## Data Availability

The original contributions presented in the study are included in the article/Supplementary material, further inquiries can be directed to the corresponding authors.
