# Supplementary material for: Association of ultra-processed food consumption with all-cause and cause-specific mortality: population-based cohort study
Source: Front Nutr. 2026 May 28;13:1820451. doi: 10.3389/fnut.2026.1820451 (PMC13253435; doi:10.3389/fnut.2026.1820451)
Supplement: Supplementary file 1 [file Data_Sheet_1.PDF]

Supplementary figures and tables

Association of ultra-processed food consumption with all cause and cause specific mortality: population based cohort study

Supplementary Figure 1. Distribution of the main exposure (proportion of ultra-processed food in the diet) in the whole population.

Supplementary Figure 2. Relative contribution of each food group to ultra-processed food consumption in diet.

Supplementary Table 1. Comparison of baseline characteristics between excluded and included population.

Supplementary Table 2. Ultra-processed foods in each food group and energy values assigned to 64 food items of the diet history questionnaire.

Supplementary Table 3. Distribution of variables with missing values before and after imputation.

Supplementary Table 4. Hazard ratios and 95% confidence intervals for mortality according to quarters of subgroups of ultra-processed food consumption.

Supplementary Table 5. Subgroup analyses on the association between ultra-processed food consumption and all cause mortality.

Supplementary Table 6. Sensitivity analyses on the association between proportion of ultra-processed food in the diet and all cause mortality.

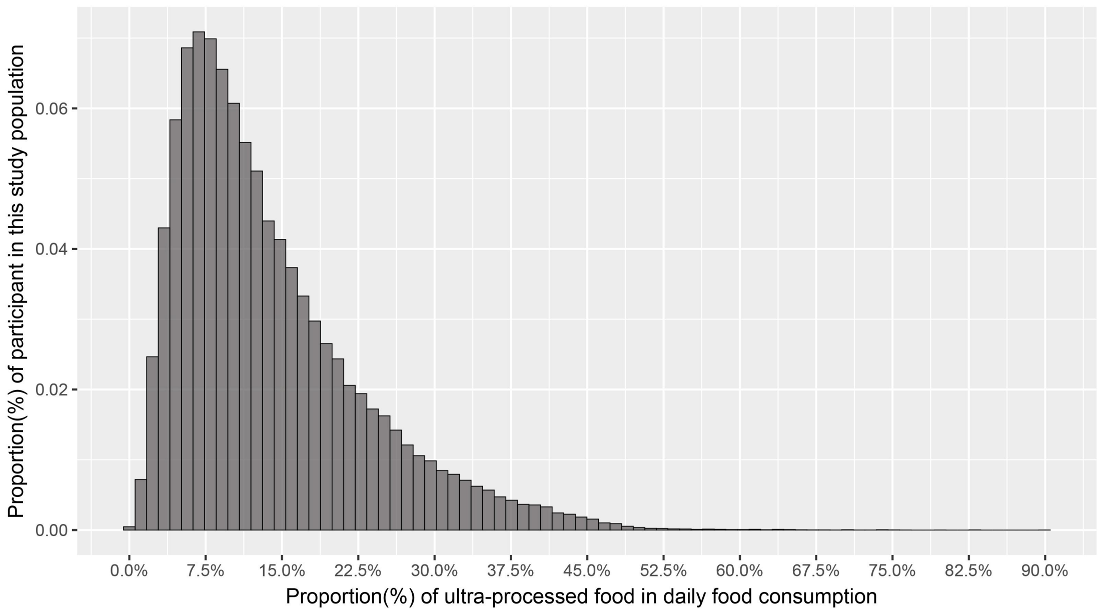

Supplementary Figure 1. Distribution of the main exposure (proportion of ultra-processed food in the diet) in the whole population.

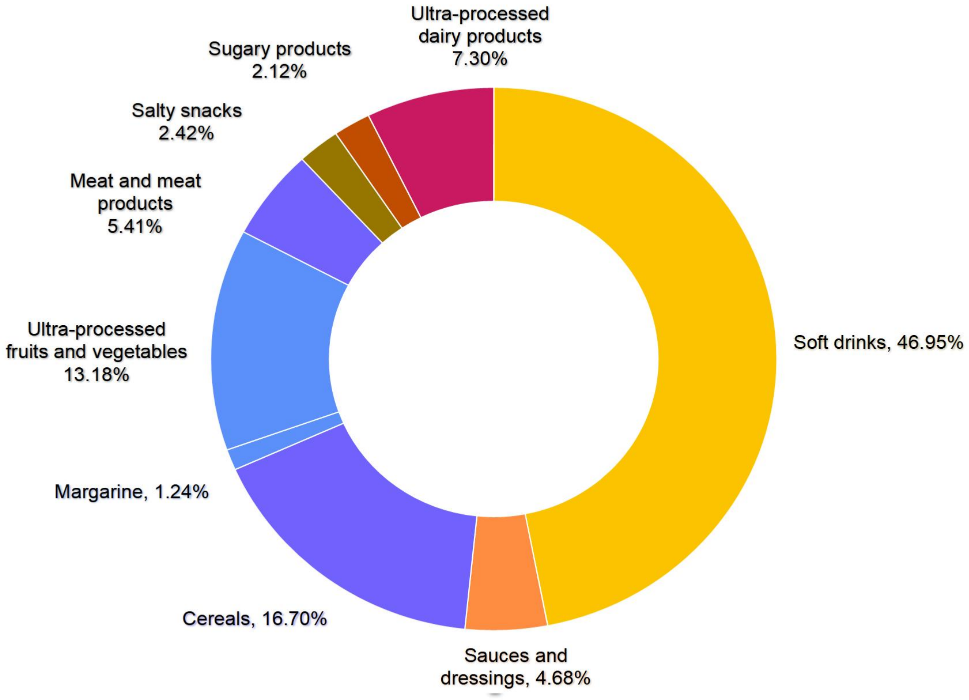

Supplementary Figure 2. Relative contribution of each food group to ultra-processed food consumption in diet.

Supplementary Table 1. Comparison of sociodemographic characteristics between excluded and included populations [1]

Sociodemographic characteristics	Excluded population	Included population	Standardized difference
Number of participants	68666	86221	
Age, years	67.00 ± 5.87	65.26 ± 5.68	0.45
Sex			0.01
Male	37184 (54.2)	39494 (45.8)	
Female	31482 (45.8)	46727 (54.2)	
Educational degree			
Some college or less	42901 (67.6)	54089 (62.9)	0.01
College graduate	9892 (15.6)	15450 (18.0)	
Postgraduate	10713 (16.9)	16514 (19.2)	
Occupation			
Working	23135 (46.0)	36090 (42.0)	0.08
Homemaker	6412 (10.1)	10574 (12.3)	
Retired	29136 (46.0)	35372 (41.2)	
Other	4696 (7.4)	3793 (4.4)	
Smoking status			
Current	8040 (12.6)	8013 (9.3)	0.45
Former	28888 (45.3)	35727 (42.4)	
Never	26798 (42.1)	42470 (49.3)	
Number of cigarettes smoked per day			
0	26798 (42.1)	42470 (49.3)	0.90
1-10	8853 (13.9)	11777 (13.7)	
11-20	13051 (20.5)	16291 (18.9)	
21-30	7398 (11.6)	8629 (10.0)	
31-40	4402 (6.9)	4438 (5.2)	
41-60	2505 (3.9)	2066 (2.4)	
61-80	467 (0.7)	377 (0.4)	
>80	144 (0.2)	76 (0.1)	
Body mass index [2]	27.72± 5.20	26.96 ± 4.67	0.34
Family history of cancer	34955 (55.1)	48365 (56.2)	0.34
History of hypertension	26253 (41.4)	24727 (28.8)	0.39
Trial group			
Screening group	33269 (48.5)	44174 (51.2)	0.82
Control group	35397 (51.5)	42047 (48.8)	

[1] Values are mean ± standard deviation or counts (percentage) as indicated. Standardized difference was calculated using the formula available in the paper by Austin PC. (DOI: 10.1080/00273171.2011.568786).

[2] Weight (kg)/height (m)

Supplementary Table 2. Ultra-processed foods in each food group and energy values assigned to 64 food items of the diet history questionnaire

Food group	Ultra-processed foods	Energy per 100 g (kcal)
Soft Drinks	Soft drinks, diet/caffeinated	49
	Soft drinks, regular/caffeinated	49
	Soft drinks, diet/decaffeinated	40
	Soft drinks, regular/decaffeinated	40
Sauces and dressings	Gravy	53
	Cheese sauce	160
	Salad dressing, low fat on salad and vegetables	160
	Salad dressing, nonfat on salad and vegetables	107
	Salad dressing, regular on salad and vegetables	430
	Mayonnaise, diet on salad	250
	Mayonnaise, fat free on salad	64
	Mayonnaise, regular on salad	680
	Mayonnaise, diet on sandwich	250
	Mayonnaise, fat free on sandwich	64
	Mayonnaise, regular on sandwich	680
Cereals	English muffins and bagels	227
	White bread/rolls	266
	Corn bread/muffins	305
	Biscuits	324
	Quick breads	297
	Donuts, sweet rolls, Danishes, and pop tarts	321
	Pancakes, waffles, and French toast	270
	Ready-to-eat cereal, highly fortified	374
	Ready-to-eat cereal, other	374
	Cookies and brownies	405
	Cakes, low fat	283
	Cakes, regular	389
	Pies, cream/custard/other	210
	Pies, fruit	209
	Pies, pecan	407
	Pies, pumpkin/sweet potato	260
	Lasagna, ravioli, shells	177
	Macaroni and cheese	221
	Pizza without meat	233
Margarine	Margarine, diet	533
	Margarine, fat free	330
	Margarine, regular	533
	Margarine, diet on bread	533
Ultra-processed fruits and vegetables	Fried potatoes	196
	Potato salad	173
	Fruit drinks, diet	1
	Fruit drinks, regular	10
Meat and meat products	Restaurant or industrial hamburgers	244

	Cold cuts	164
	Lunch meat	251
	Pizza with meat	277
	Hot dogs, turkey/low fat	127
	Hot dogs, regular	332
	Sausages	325
Salty snacks	Crackers	430
	Potato/corn/other chips	532
	Potato/corn/other chips-low fat	482
	Popcorn	530
Sugary products	Candy, chocolate	462
	Candy, not chocolate	394
	Saccharine in coffee and tea	360
Ultra-processed dairy products	Sour cream, low fat	181
	Sour cream, regular	198
	Cream cheese, low fat	201
	Cream cheese, regular	350
	Ice cream/ice milk, low fat	207
	Regular ice cream	216
	Frozen yogurt, ices, and sorbet	72
	Creamed soups	46

Supplementary Table 3. Distribution of variables with missing values before and after imputation [1]

Covariates	Before imputation	After imputation	Number (%) with missing values
Educational level			
Some college or less	54089 (62.86)	54257 (62.93)	168 (0.19)
College graduate	15450 (17.95)	15450 (17.92)	
Postgraduate	16514 (19.19)	16514 (19.15)	
Smoking status			
Current	8013(9.29)	8013(9.29)	11 (0.01)
Former	35727 (41.44)	35727 (42.44)	
Never	42470 (49.26)	42481 (49.27)	
Number of cigarettes smoked per day			
0	42470 (49.31)	42470 (49.37)	97(0.11)
1-10	11777 (13.67)	11777 (13.66)	
11-20	16291 (18.92)	16291 (18.89)	
21-30	8629 (10.02)	8629 (10.01)	
31-40	4438 (5.15)	4438 (5.15)	
41-60	2066 (2.40)	2066 (2.40)	
61-80	377 (0.44)	377 (0.44)	
>80	76 (0.08)	76 (0.08)	
Family history of any cancer	48365 (56.2)	48365 (56.2)	233 (0.27)
History of hypertension	24727 (28.67)	24727 (28.67)	493 (0.57)
Body mass index [2]	26.96± 4.67	26.95± 4.64	1125 (1.30)
Physical activity level (min/week) [3]	126.55±122.44	126.72±122.71	20478 (23.75)

[1] n = 86,221. Values are mean (standard deviation) or counts (percentage) as indicated.

[2] Weight (kg)/height (m)^2.

[3] Total time of moderate-to-vigorous physical activity per week

Supplementary Table 4 Hazard ratios and 95% confidence intervals for mortality according to quarters of subgroups of ultra-processed food consumption.

	Sex specific quarters of proportion of ultra-processed food consumption[1]				
	**Q1** (n = 21,556)	**Q2** (n = 21,555)	**Q3** (n = 21,554)	**Q4** (n = 21,556)	***P*** **trend**
**All-case mortality**					
Cereals	1.00 (reference)	0.97 (0.93-1.00)	0.99 (0.96-1.03)	1.05 (1.01-1.10)	0.005
Soft drinks	1.00 (reference)	0.98 (0.95-1.02)	0.98 (0.94-1.01)	1.05 (1.02-1.09)	0.011
Sauces and dressings	1.00 (reference)	0.94 (0.92-0.98)	0.93 (0.89-0.96)	0.94 (0.91-0.98)	0.001
Meat and meat products	1.00 (reference)	1.02 (0.98-1.06)	0.98 (0.95-1.01)	1.02 (0.98-1.06)	0.678
Salty snacks	1.00 (reference)	0.93 (0.90-0.96)	0.90 (0.86-0.93)	0.87 (0.83-0.90)	<0.001
Ultra-processed dairy products	1.00 (reference)	0.95 (0.91-0.98)	0.95 (0.91-0.99)	0.95 (0.90-1.00)	0.692
Margarine	1.00 (reference)	1.00 (0.97-1.04)	1.01 (0.97-1.04)	0.99 (0.95-1.03)	0.690
Sugary products	1.00 (reference)	0.93 (0.89-0.96)	0.91 (0.88-0.95)	0.94 (0.90-0.97)	0.002
Ultra-processed fruits and vegetables	1.00 (reference)	0.92 (0.89-0.96)	0.98 (0.95-1.02)	1.02 (0.99-1.06)	0.014
**Cancer mortality**					
Cereals	1.00 (reference)	0.96 (0.90-1.03)	1.04 (0.98-1.12)	1.04 (0.97-1.12)	0.080
Soft drinks	1.00 (reference)	1.05 (0.98-1.12)	0.98 (0.92-1.05)	1.00 (0.94-1.07)	0.596
Sauces and dressings	1.00 (reference)	0.98 (0.92-1.05)	0.97 (0.91-1.04)	1.00 (0.93-1.07)	0.902
Meat and meat products	1.00 (reference)	1.07 (1.00-1.14)	1.02 (0.95-1.09)	1.07 (0.99-1.14)	0.224
Salty snacks	1.00 (reference)	1.00 (0.93-1.06)	0.90 (0.86-0.98)	0.90 (0.84-0.97)	<0.001
Ultra-processed dairy products	1.00 (reference)	0.99 (0.92-1.06)	1.05 (0.98-1.12)	1.01 (0.91-1.11)	0.485
Margarine	1.00 (reference)	0.98 (0.92-1.05)	0.99 (0.92-1.05)	0.99 (0.93-1.06)	0.866
Sugary products	1.00 (reference)	0.99 (0.93-1.06)	1.00 (0.93-1.06)	0.98 (0.92-1.05)	0.165
Ultra-processed fruits and vegetables	1.00 (reference)	0.94 (0.88-1.00)	0.99 (0.92-1.04)	0.97 (0.91-1.04)	0.829
**Circulatory mortality**					
Cereals	1.00 (reference)	0.95 (0.89-1.02)	0.96 (0.89-1.03)	1.08 (1.00-1.16)	0.055
Soft drinks	1.00 (reference)	0.98 (0.92-1.05)	0.96 (0.90-1.03)	1.09 (1.02-1.17)	0.032
Sauces and dressings	1.00 (reference)	0.91 (0.85-0.97)	0.93 (0.87-0.99)	0.91 (0.85-0.98)	0.026
Meat and meat products	1.00 (reference)	0.99 (0.93-1.06)	1.05 (0.99-1.13)	0.99 (0.92-1.06)	0.593
Salty snacks	1.00 (reference)	0.89 (0.83-0.95)	0.88 (0.82-0.94)	0.84 (0.79-0.91)	<0.001
Ultra-processed dairy products	1.00 (reference)	0.89 (0.83-0.95)	0.90 (0.84-0.97)	0.93 (0.84-1.02)	0.153

Margarine	1.00 (reference)	1.05 (0.98-1.12)	1.02 (0.95-1.09)	0.98 (0.92-1.05)	0.503
Sugary products	1.00 (reference)	0.91 (0.86-0.98)	0.91 (0.85-0.97)	0.93 (0.87-1.00)	0.078
Ultra-processed fruits and vegetables	1.00 (reference)	0.90 (0.84-0.96)	0.98 (0.91-1.05)	1.01 (0.94-1.08)	0.298
**Nervous mortality**					
Cereals	1.00 (reference)	0.96 (0.85-1.09)	0.98 (0.86-1.12)	1.14 (1.01-1.31)	0.045
Soft drinks	1.00 (reference)	0.86 (0.76-0.97)	1.03 (0.91-1.14)	1.00 (0.88-1.14)	0.382
Sauces and dressings	1.00 (reference)	1.08 (0.95-1.22)	1.00 (0.88-1.13)	1.02 (0.89-1.16)	0.954
Meat and meat products	1.00 (reference)	0.94 (0.83-1.05)	0.90 (0.79-1.02)	1.01 (0.89-1.25)	0.991
Salty snacks	1.00 (reference)	0.92 (0.81-1.03)	0.91 (0.81-1.03)	0.84 (0.74-0.96)	0.012
Ultra-processed dairy products	1.00 (reference)	1.02 (0.90-1.15)	0.88 (0.77-1.01)	0.96 (0.81-1.14)	0.716
Margarine	1.00 (reference)	1.03 (0.91-1.17)	0.97 (0.85-1.09)	0.92 (0.81-1.04)	0.139
Sugary products	1.00 (reference)	0.81 (0.72-0.92)	0.84 (0.74-0.95)	0.94 (0.83-1.06)	0.554
Ultra-processed fruits and vegetables	1.00 (reference)	0.90 (0.79-1.01)	0.91 (0.80-1.03)	1.10 (0.97-1.24)	0.112
**Other mortality**					
Cereals	1.00 (reference)	0.98 (0.91-1.06)	0.96 (0.89-1.04)	1.03 (0.95-1.12)	0.619
Soft drinks	1.00 (reference)	0.99 (0.92-1.06)	1.05 (0.97-1.13)	1.16 (1.07-1.25)	<0.001
Sauces and dressings	1.00 (reference)	0.94 (0.87-1.01)	0.87 (0.81-0.94)	0.92 (0.85-0.99)	0.009
Meat and meat products	1.00 (reference)	0.96 (0.89-1.04)	0.96 (0.89-1.04)	1.02 (0.94-1.10)	0.726
Salty snacks	1.00 (reference)	0.91 (0.84-0.98)	0.90 (0.83-0.97)	0.85 (0.78-0.92)	<0.001
Ultra-processed dairy products	1.00 (reference)	0.97 (0.90-1.05)	0.94 (0.87-1.02)	0.97 (0.87-1.07)	0.017
Margarine	1.00 (reference)	0.99 (0.92-1.08)	1.00 (0.93-1.08)	1.02 (0.95-1.10)	0.555
Sugary products	1.00 (reference)	0.93 (0.86-1.00)	0.86 (0.80-0.93)	0.93 (0.86-1.00)	0.022
Ultra-processed fruits and vegetables	1.00 (reference)	0.91 (0.84-0.98)	1.00 (0.93-1.08)	1.05 (0.97-1.14)	0.035

1 Adjusted for age (years), sex (male, female), race (non-Hispanic White, non-Hispanic Black, Hispanic, and other race/ethnicity), and marital status， smoking status [current (>20 cigarettes/day, 10-20 cigarettes/day, <10 cigarettes/day), former (stop smoking >15 years, stop smoking ≤15 years), never], alcohol consumption (g/day), body mass index (kg/m^2), aspirin use (yes, no), history of hypertension (yes, no), family of cancer (yes, no), energy intake from diet (kcal/day), physical activity, and educational level.

Supplementary Table 5. Subgroup analyses on the association between proportion of ultra-processed food in the diet (%) and total mortality.

Subgroup variable	Number of cases	Ultra-processed food consumption consumption		
		Quartile 1	Quartile 4	*P* for interaction
**Age at diet history questionnaire completion (years)**				
≥65		1.00 (reference)	1.05 (1.00, 1.10)	0.219
<65		1.00 (reference)	0.98 (0.91, 1.05)	
**Sex**				
Males		1.00 (reference)	1.07 (1.02, 1.13)	0.221
Females		1.00 (reference)	1.17 (1.11, 1.24)	
**Body mass index (kg/m^2)**				
≥25		1.00 (reference)	1.16 (1.11, 1.22)	0.011
<25		1.00 (reference)	1.06 (0.99, 1.13)	
**Smoking status**				
Current or former smokers stopping smoking ≤15 years		1.00 (reference)	1.18 (1.12, 1.23)	0.091
Never or former smokers stopping smoking >15 years		1.00 (reference)	1.05 (0.98, 1.12)	
**Alcohol consumption**				
≥ median		1.00 (reference)	1.09 (1.03, 1.16)	0.695
< median		1.00 (reference)	1.05 (1.00, 1.11)	
**Trial group**				
Screening group		1.00 (reference)	1.13 (1.07, 1.19)	0.397
Control group		1.00 (reference)	1.09 (1.03, 1.15)	

Supplementary Table 6. Sensitivity analyses on the association between proportion of ultra-processed food in the diet (%) and the total mortality [a]

Categories	Sample size	Sex specific quarters of proportion of ultra-processed food consumption				
		Quartile 1	Quartile 2	Quartile 3	Quartile 4	*P* for trend
**Additional adjustment on model 2**						
Healthy Eating Index-2015	86221	1.00 (reference)	0.96 (0.93, 1.00)	0.99 (0.96, 1.03)	1.04 (1.00, 1.08)	0.029
Intakes of fruit, vegetable, red and white meat and whole grain	86221	1.00 (reference)	0.99 (0.96, 1.03)	1.05 (1.02, 1.09)	1.13 (1.08, 1.17)	< 0.001
Intakes of protein, fat, sodium, carbohydrates, and dietary fiber	86221	1.00 (reference)	0.98 (0.94, 1.01)	1.02 (0.98, 1.06)	1.07 (1.03, 1.12)	< 0.001
Excluded cases occurred within the first two years of follow-up	86152	1.00 (reference)	0.99 (0.96, 1.03)	1.05 (1.01, 1.09)	1.13 (1.09, 1.17)	< 0.001
Excluded cases occurred within the first three years of follow-up	86077	1.00 (reference)	1.00 (0.96, 1.03)	1.05 (1.01, 1.09)	1.13 (1.09, 1.17)	< 0.001
Excluded cases occurred within the first five years of follow-up	85587	1.00 (reference)	0.99 (0.96, 1.03)	1.04 (1.00, 1.08)	1.12 (1.08, 1.17)	< 0.001
Repeated the analysis with quarters of ultra-processed food intake (in % kcal/day)	86221	1.00 (reference)	0.98 (0.94, 1.01)	1.00 (0.97, 1.04)	1.07 (1.04, 1.12)	< 0.001
Repeated the analysis with quarters of ultra-processed food intake (quantity)	86221	1.00 (reference)	0.97 (0.94, 1.01)	1.00 (0.97, 1.04)	1.10 (1.06, 1.15)	< 0.001
Repeated the analysis with quarters of proportion of ultra-processed food consumption	86221	1.00 (reference)	1.02 (0.98, 1.06)	1.06 (1.02, 1.10)	1.14 (1.09, 1.18)	< 0.001

[a] Adjusted for age (years), sex (male, female), race (non-Hispanic White, non-Hispanic Black, Hispanic, and other race/ethnicity), and marital status， smoking status [current (>20 cigarettes/day, 10-20 cigarettes/day, <10 cigarettes/day), former (stop smoking >15 years, stop smoking ≤15 years), never], alcohol consumption (g/day), body mass index (kg/m^2), aspirin use (yes, no), history of hypertension (yes, no), family of cancer (yes, no), energy intake from diet (kcal/day), physical activity, and educational level.
