# Supplementary material for: Association of ultra-processed food consumption with all-cause and cause-specific mortality: population-based cohort study
Source: Front Nutr. 2026 May 28;13:1820451. doi: 10.3389/fnut.2026.1820451 (PMC13253435; doi:10.3389/fnut.2026.1820451)
Supplement: Supplementary file 2 [file Data_Sheet_2.pdf]

Materials and Methods

Study Population

The Prostate, Lung, Colorectal, and Ovarian (PLCO) Cancer Screening Trial, which was a randomized multicenter controlled study, was aimed to determine whether screening exams or tests could reduce the risk of mortality from prostate, lung, colorectal and ovarian cancers. Study design and methodology of the PLCO trial have been reported in detail elsewhere[1]. Approximately 155,000 individuals aged 55-74 years in this trial during the period from 1993 to 2001 from 10 screening centers in the United States. Participants were enrolled and randomly assigned to the screening arm and control arm in equal proportions. Individuals in control arm received the usual care, whereas those in screening arm received a cancer screening intervention, including prostate-specific antigen (PSA) testing and digital rectal exams for prostate cancer screening, posteroanterior chest X-ray to screen lung cancer, and the flexible microscopy to screen colorectal cancer. The PLCO trial was approved by the US National Cancer Institute and the Institutional Review Board of each screening center. All of the participants provided their informed consents[2].

In this study, due to the fact that the outcome was prostate cancer, the considering study population was only men. The following males were further excluded: 1) overall 2,824 males without returning a baseline questionnaire; 2) overall 17,510 males without completing a Dietary History Questionnaire (DHQ); 3) overall 2,622 with providing an invalid DHQ. A valid DHQ referred to the presence of having a DHQ completion date, DHQ completion date before death date, <8 missing DHQ items, and the absence of extreme values of calorie intake (lowest or highest 1%). Notably, the above-mentioned criteria were jointly defined by nutritionists, epidemiologists, and statisticians from the US National Cancer Institute. 4) Overall 4,194 males diagnosed with cancer before DHQ completion; 5) those with prostate cancer diagnosed or dead or without annual study update ≤1 y after study entry (n=97); and 6) overall 7 males with outcome events observed between trial entry and DHQ completion (outcome events referred to loss to follow-up, death or incident prostate cancer). After these exclusions, a total of 82,221 participants were included in

this cohort. Moreover, we compared the populations between inclusion and exclusion; the standardized differences were found to be < 0.1, which indicated that the possibility of non-participation bias was small because of the exclusion of numerous participants (Supplementary Table 1).

Assessment of ultra-processed food consumption

All food and beverage items of the DHQ composition table classified into one of the four NOVA food groups by two researchers (YDL and TW) using the method described in the literature[3]. The NOVA system categorizes foods according to the degree of industrial processing as: (1) unprocessed or minimally processed foods, (2) processed culinary ingredients, (3) processed foods, and (4) ultra-processed foods [4]. In this study, we focused on the “ultra-processed foods” NOVA group, for example, beverage, sauce, and fast-food hamburgers. All ultra-processed foods were further divided into nine food groups, which is soft drinks, cereals, ultra-processed fruits and vegetables, ultra-processed dairy products, meat and fish, sauces and dressings, salty snacks, sugary products, and margarine[3]. Detailed definitions and examples of UPF items are provided in Supplementary Table 2.

Assessment of covariates

A sex-specific baseline questionnaire solicited information on age, race, weight, height, marital status, education, physical activity, smoking status, family history of cancer and history of hypertension, and other factors. Categorical variables were presented as percentages of the total, and continuous variables were expressed as mean ± standard deviation. DHQ was used to collect dietary information[5]. Age at DHQ completion and alcohol intake were collected through this questionnaire. Daily food intake (g/day) was estimated by multiplying reported frequency by portion size, and energy and nutrient intakes were computed using the DietCalc analysis program (National Cancer Institute, Bethesda, MD), which incorporates national nutrient databases. Diet quality was evaluated using the Healthy Eating Index-2015, as described previously[6]. Physical activity, defined as the total time spent in moderate-to-vigorous exercise per week, was assessed using a self-administered supplemental questionnaire. In the multivariable Cox models, continuous variables

(e.g., age, BMI, energy intake) were modeled as continuous. Categorical variables (e.g., education, smoking status, family history of cancer, and history of hypertension) were included with categories as defined in Supplementary Table 1. The categorization methods for all variables are detailed in Supplementary Table 1.

Ascertainment of outcomes

The indication of death certifcate and family report was additional sources for the ascertainment. The primary outcome of this study was all cause mortality. The secondary outcomes included deaths from cancer (ICD-9 codes 140-207), circulatory diseases (ICD-9 codes 390-459, 430-438), diseases of the nervous system (ICD-9 codes 320-389) and other diseases (ICD-9 codes 460-519, 240-279, 001-139, 580-629, 800-999).

Statistical analysis

In this study, the continuous variables were expressed as means with standard deviation, and categorical variables are expressed as percentages. Education was categorized as postgraduate, college, college below. Race/ethnicity was categorized as categorical variable of Hispanic White, non-Hispanic Black, Hispanic, and Asian. Other categorical variables included smoking status, classified as current, former and never, family history of cancer, history of hypertension. Continuous variables included age, BMI (kg/m2), energy intake (kcal/day), alcohol consumption (g/day), food consumption, nutrient intake, physical activity (min/week), and Healthy Eating Index-2015. For all covariates except physical activity, 5% or less of values were missing and were imputed to the modal value (for categorical variables) or median (for continuous variables)[7]. For physical activity, the proportion of missing values was higher (25.56%) and these values were considered as missing at random and then multiple imputation with chained equations was used to impute them (the number of imputations set at 25)[8]. A missing data was included into the models for this variable because massive imputation for a non-negligible number of participants and risk of selection bias was considered. We further conduct main analyses in participants with complete data for comparison. The corresponding distribution of variables with missing values before and after data imputation was present in

Supplementary Table 3. The differences in participants baseline characteristics between quarters of the ultra-processed food consumption were examined by using analysis of Kruskal-Wallis rank sum test or $\chi 2$ tests wherever appropriate. We used Cox proportional hazards models with person-year as the primary timescale to evaluate the association between the proportion of ultra-processed foods in the diet (coded as a continuous variable or as sex specific quarters) and all-cause mortality and cause specific mortality. In these models, participants contributed person time from the date of DHQ completion to the date of diagnosis of RCC, the date of death, or 31 December 2009, whichever occurred first (Figure 2). Ultra-processed food consumption was divided into quartile. We estimated hazard ratios and 95% confidence intervals with the lowest quarter as the reference category. In models based on sex quarters of ultra-processed foods consumption, we tested for linear trend by coding the median value of each quarter of ultra-processed food as ordinal variable.

Covariates were selected on the basis of our causal knowledge from previous literature instead of the statistical criteria[9]. In the multivariable model, we adjusted for age at DHQ completion, race/ethnicity, marital status, age at DHQ completion, race/ethnicity, body mass index (BMI, continuous), alcohol consumption (g/day, continuous), energy intake (kcal/day, continuous), family history of cancer, and history of hypertension. The proportional hazards assumption was assessed using Schoenfeld residuals[10]. We also tested for the dose-response relation by using the restricted cubic spline regression[2].

Subgroup analyses were conducted to determine whether the observed associations between ultra-processed food consumption and all cause morality were modified by age at DHQ completion (≥65 vs. <65 years), BMI (≥25 vs. <25), smoking status (current or former smokers stopping smoking ≤15 years vs never or former smokers stopping smoking >15 years), trial group (screening compared with control groups), and alcohol consumption (≥median vs <median). A *P* value for interaction was obtained by comparing models with and without interaction terms before performing the above-mentioned subgroup analyses to avert the possibly

spurious subgroup differences. Furthermore, to evaluate the independent and combined association of ultra-processed food intake and overall diet quality with mortality, we jointly categorized participants by quarters of the Healthy Eating Index-2015 (HEI-2015) and ultra-processed food intake, using those in the highest HEI and lowest ultra-processed food quartiles as the reference group.

Sensitivity analyses were performed to evaluate the robustness of our results: (1) excluding deaths occurring within the first 2, 3, or 5 years of follow-up to avoid reverse causality bias; (2) adjustment for several indicators of diet quality, including Healthy Eating Index-2015, intakes of fruit, vegetable, red and white meat, whole grain protein, fat, sodium, carbohydrates, and dietary fiber; (3) repeating main analyses with using daily percentage energy intake of ultra-processed food consumption; (4) repeating main analyses with using daily the quantity of ultra-processed food consumption.

We did an analysis to examine the association between the aforementioned nine individual food groups of ultra-processed food consumption and all cause and cause specific mortality and the main contributor(s) to this association could be determined. All statistical analyses were performed using R software (version 4.3.1) and STATA (version 16.0). Two-sided $p < 0.05$ was considered statistically significant.

Reference

1. Prorok, P.C., et al., *Design of the Prostate, Lung, Colorectal and Ovarian (PLCO) Cancer Screening Trial.* Control Clin Trials, 2000. 21(6 Suppl): p. 273s-309s.
2. Li, Y.D., et al., *Ultra-processed food consumption and renal cell carcinoma incidence and mortality: results from a large prospective cohort.* BMC Med, 2024. 22(1): p. 459.
3. Zhong, G.C., et al., *Ultra-processed food consumption and the risk of pancreatic cancer in the Prostate, Lung, Colorectal and Ovarian Cancer Screening Trial.* Int J Cancer, 2023. 152(5): p. 835-844.
4. Monteiro, C.A., et al., *Ultra-processed foods: what they are and how to identify them.* Public Health Nutr, 2019. 22(5): p. 936-941.
5. Subar, A.F., et al., *Comparative validation of the Block, Willett, and National Cancer Institute food frequency questionnaires : the Eating at America's Table Study.* Am J Epidemiol, 2001. 154(12): p. 1089-99.
6. Krebs-Smith, S.M., et al., *Update of the Healthy Eating Index: HEI-2015.* J Acad Nutr Diet, 2018. 118(9): p. 1591-1602.
7. Srour, B., et al., *Ultra-processed food intake and risk of cardiovascular disease: prospective*

cohort study (NutriNet-Santé). Bmj, 2019. 365: p. l1451.
8. Spratt, M., et al., *Strategies for multiple imputation in longitudinal studies.* Am J Epidemiol, 2010. 172(4): p. 478-87.
9. Hernán, M.A., et al., *Causal knowledge as a prerequisite for confounding evaluation: an application to birth defects epidemiology.* Am J Epidemiol, 2002. 155(2): p. 176-84.
10. Hua, K., et al., *Network Meta-Analysis With Individual Participant-Level Data of Time-to-Event Outcomes Using Cox Regression.* Stat Med, 2025. 44(5): p. e70027.
